# Identification of candidate genes and pathways associated with juvenile idiopathic arthritis by integrative transcriptome-wide association studies and mRNA expression profiles

**DOI:** 10.1186/s13075-023-03003-z

**Published:** 2023-02-08

**Authors:** Ruoyang Feng, Mengnan Lu, Chunyan Yin, Ke Xu, Lin Liu, Peng Xu

**Affiliations:** 1 Department of Joint Surgery, HongHui Hospital, Xian Jiaotong University, Xi’an, 710054 Shanxi China; 2 Department of Pediatrics, The Second Affiliated Hospital of Xi’an Jiaotong University, Xi’an, Shaanxi China

**Keywords:** Juvenile idiopathic arthritis, GWAS, TWAS, Environment factors, Susceptibility gene

## Abstract

**Aim:**

Juvenile idiopathic arthritis (JIA) is the most common chronic rheumatic disease of childhood, with genetic susceptibility and pathological processes such as autoimmunity and autoinflammation, but its pathogenesis is unclear. We conducted a transcriptome-wide association study (TWAS) using expression interpolation from a large-scale genome-wide association study (GWAS) dataset to identify genes, biological pathways, and environmental chemicals associated with JIA.

**Methods:**

We obtained published GWAS data on JIA for TWAS and used mRNA expression profiling to validate the genes identified by TWAS. Gene Ontology (GO) and Kyoto Encyclopedia of Genes and Genomes (KEGG) pathway enrichment analyses were performed. A protein–protein interaction (PPI) network was generated, and central genes were obtained using Molecular Complex Detection (MCODE). Finally, chemical gene expression datasets were obtained from the Comparative Toxicogenomics database for chemical genome enrichment analysis.

**Results:**

TWAS identified 1481 genes associated with JIA, and 154 differentially expressed genes were identified based on mRNA expression profiles. After comparing the results of TWAS and mRNA expression profiles, we obtained eight overlapping genes. GO and KEGG enrichment analyses of the genes identified by TWAS yielded 163 pathways, and PPI network analysis as well as MCODE resolution identified a total of eight clusters. Through chemical gene set enrichment analysis, 287 environmental chemicals associated with JIA were identified.

**Conclusion:**

By integrating TWAS and mRNA expression profiles, genes, biological pathways, and environmental chemicals associated with JIA were identified. Our findings provide new insights into the pathogenesis of JIA, including candidate genetic and environmental factors contributing to its onset and progression.

**Supplementary Information:**

The online version contains supplementary material available at 10.1186/s13075-023-03003-z.

## Introduction

Juvenile idiopathic arthritis (JIA) is an autoimmune disease characterized by chronic inflammation of the joints, encompassing all forms of chronic inflammatory arthritis of unknown causes and having an onset before the age of 16 years [1]. The reported prevalence varies between 16 and 150 per 100,000 individuals [2]. It typically lasts for longer than 6 months with arthritis present for at least 6 weeks [3, 4]. Joint involvement usually starts with synovitis and the formation of inflammatory tissue, called the pannus, which destroys hyaline cartilage, erodes the bone, and leads to articular destruction and ankylosis [5]. It has been estimated that 37–63% of adults diagnosed with JIA as children maintain active disease [6]. Additionally, children with JIA are at a significant risk for cardiovascular disease in childhood [7]. Although little is known regarding the underlying mechanism, genetic factors play an important role in the pathogenesis of autoimmune diseases [8]. Therefore, studies of the genetic basis of JIA are necessary to provide a basis and new directions for prevention, early diagnosis, and targeted treatment.

JIA is believed to have a complex molecular basis and is influenced by both genetic and environmental factors [9]. Advances in genetic techniques have prompted research on the genetic basis of JIA, including genome-wide association studies (GWAS), which are a powerful approach to identify genetic loci associated with polygenic complex diseases and traits [10]. However, GWAS is limited in assessing the risk of complex diseases because most single nucleotide polymorphisms identified by this approach are located in non-coding regions [11]. Transcriptome-wide association studies (TWAS) show great promise in interpreting GWAS signatures and are powerful in detecting associations between gene expression levels and complex diseases [12]. TWAS can be used to integrate expression quantitative trait locus (QTL) data with GWAS to identify genes whose regulation is associated with the disease risk [13] and to identify complex trait associations [14]. For example, Gusev et al. used TWAS to identify 69 novel genes in blood and adipose tissue associated with obesity-related traits [15].

Environmental risk factors are strongly associated with the development of autoimmune diseases. In individuals at an increased genetic risk for a disease, environmental or lifestyle factors can lead to early alterations in the immune system and the disruption of self-tolerance, ultimately leading to overt disease [16]. There is strong evidence that chemicals produce biological effects by affecting gene expression. For example, previous studies have revealed that altered gene expression levels in peripheral blood mononuclear cells are associated with occupational benzene exposure [17]. Furthermore, the altered composition of gut microbes, which are affected by environmental conditions, has been implicated in JIA pathogenesis [18]. It has also been shown that sulfur dioxide (SO_2_) from atmospheric pollution increases the rate of JIA [19]. Therefore, analyzing the effects of chemicals on JIA is crucial.

**Fig. 1:**
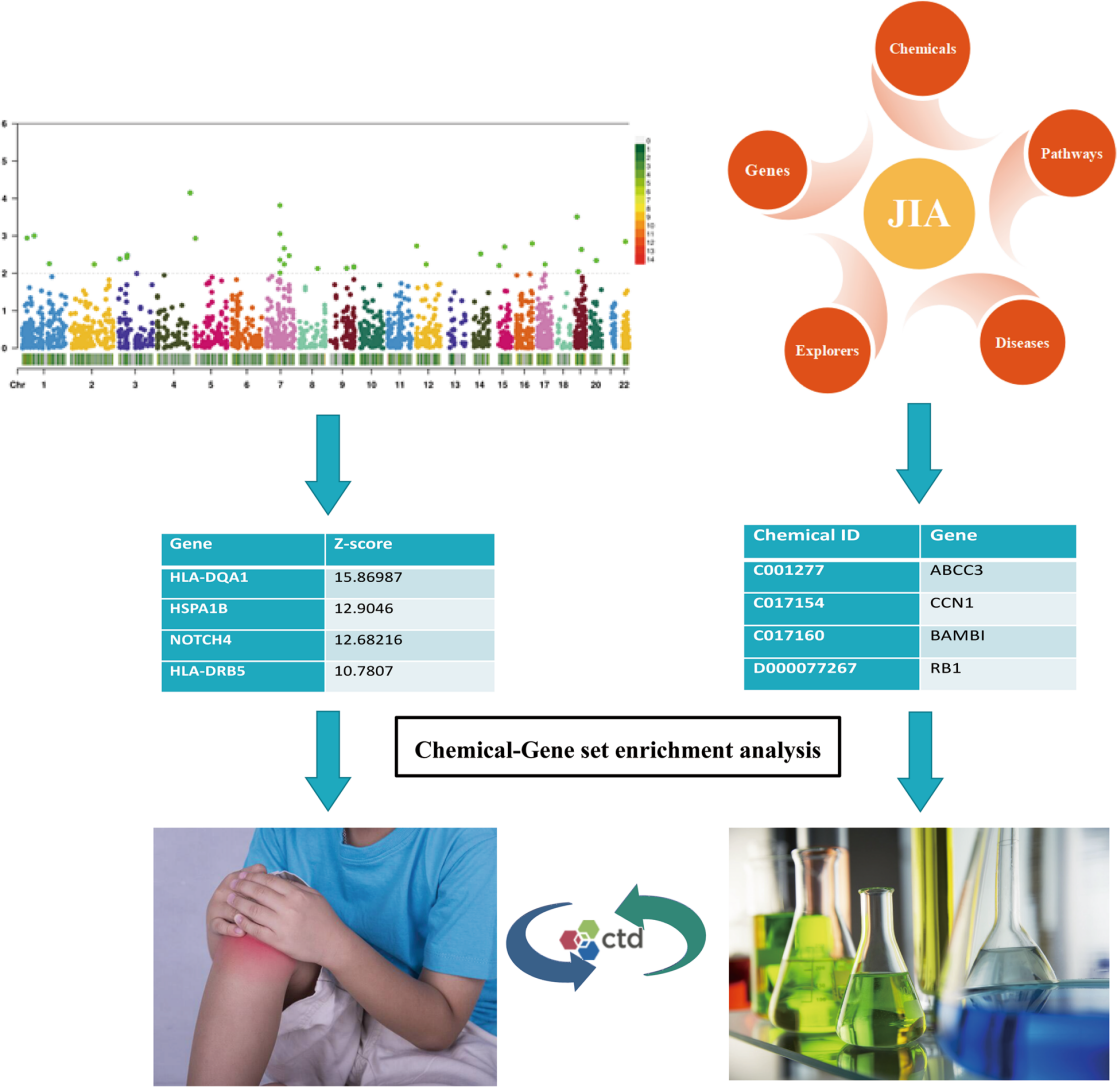
The research design and general process of this research

In this study, candidate genes and biological pathways associated with JIA were identified to improve our understanding of the pathogenesis of this disease. Furthermore, we examined the associations between chemicals and JIA based on chemical–gene interaction networks. An overview of the study is provided in Fig. 1.

## Data and methods

### GWAS data for JIA

GWAS data for JIA were obtained from the literature [20]. In brief, Elena et al. evaluated 4520 UK JIA samples and 9965 samples from healthy individuals using Illumina Infinium CoreExome and Infinium OmniExpress genotyping arrays. Finally, 12,501 individuals were retained in the QC-filtered dataset (3305 cases and 9196 healthy controls). Haplotype phasing and interpolation were performed with the Michigan Interpolation Server using SHAPEIT2 and Minimac3 as well as the Haplotype Reference Consortium reference panel. Simple linear regression using additive genetic models was used to test for genetic associations. Detailed sample characteristics, experimental design, quality control, and statistical analyses are described previously [20].

### TWAS of JIA

Common approaches for TWAS (e.g., PrediXcan, TWAS-FUSION, and SMR) can be viewed as forms of instrumental variable analyses with an emphasis on testing causal relationships between gene expression and complex traits [21]. In the present study, FUSION was used to analyze aggregated GWAS data for a TWAS of JIA (http://gusevlab.org/projects/fusion/). FUSION is a set of tools used to evaluate the association between gene expression and a target disease/phenotype based on pre-calculated gene expression weights and GWAS summary data [15]. Briefly, we used the predictive model implemented in FUSION to calculate the gene expression by combining tissue-specific expression weights with aggregated GWAS results to translate single genetic variant–phenotype associations into gene/transcript–phenotype associations for quantitative evaluations of associations. Gene expression weight panels for precomputation were downloaded from the FUSION website (http://gusevlab.org/projects/fusion/). All *P* values were then corrected for multiple testing using the Benjamini–Hochberg procedure to collect *Q* values, which represent the minimum false discovery rate (FDR) threshold at which exposure is considered significant. In our study, genes with FDR.P < 0.05 and MODELCV.R2 ≥ 0.01 were considered significant.

### Gene expression profiles for JIA

Gene profiles were downloaded from the Gene Expression Omnibus database (https://www.ncbi.nlm.nih.gov/geo/). The keywords for inclusion were (1) juvenile idiopathic arthritis, (2) *Homo sapiens*, and (3) peripheral blood tissue. Datasets with pharmacological stimulation or other interventions were excluded. Finally, we selected two datasets that met the criteria, GSE7753 [22] and GSE11083 [23]. The platform used for both chip datasets was GPL570, Affymetrix Human Genome U133 Plus 2.0 Array. The datasets involved 31 patients with JIA and 45 healthy controls. After removing inter-batch effects using the R package “sva” and the combat function [24], a differential gene expression analysis was performed using the “limma” package. Genes with |log2FC| > 1 and adjusted *p*-value < 0.05 were screened as differentially expressed genes (DEGs) in JIA. The results were visualized using the “ggplot” package. The “ComplexHeatmap” package was used to generate a heatmap [25].

### Chemical gene expression annotation dataset

A chemical gene expression annotation dataset was downloaded from the Comparative Toxicology Genomics Database (CTD) (http://ctdbase.org/downloads/), an innovative digital ecosystem that relates toxicological information for chemicals, genes, phenotypes, diseases, and exposure to advance our understanding of human health [26]. CTD integrates four main datasets, namely chemical gene interaction functions, chemical disease associations, genetic disease associations, and chemical element phenotype associations, to automatically construct a hypothetical chemical–gene–phenotype–disease network [27]. A dataset of 1,788,149 chemical–gene pairs annotated with related terms for humans and mice was used by Cheng et al. to generate a set of 11,190 chemical-associated genes [28].

### Chemically related gene set enrichment analysis (CGSEA)

A CGSEA was performed to assess the association between chemicals and complex diseases. Briefly, genome-wide pooled data (TWAS pooling) were used to explore the relationship between chemicals and many complex diseases from a genomic perspective. CTD chemical–gene interaction networks and pooled TWAS data were subjected to the weighted Kolmogorov–Smirnov tests to explore the relationships between chemicals and JIA [29]. In particular, 10,000 permutations were generated to obtain the empirical distribution of GSEA statistics for each chemical substance, and the *p*-value was calculated for each chemical substance based on the empirical distribution of CGSEA statistics. Based on the literature, we excluded genomes containing fewer than 10 or more than 500 genes to control for the effect of genome size [30]. The analysis method has been described in detail in a previous study [28].

### Functional enrichment analysis

Kyoto Encyclopedia of Genes and Genomes (KEGG) [31] and Gene Ontology (GO) [32] enrichment analyses of genes identified by the TWAS were performed to identify JIA-related biological processes. Enrichment analyses were performed using the R packages “org.Hs.eg.db” and “clusterProfiler” (https://www.R-project.org/).

### Protein–protein interaction network analysis

Protein–protein interaction (PPI) networks were generated using the STRING v11.5 database (STRING, https://string-db.org), requiring a confidence level of 0.15 and generating “active interaction sources” based on a previous study [33]. Cytoscape [34] was used to visualize interaction networks and the plugin Molecular Complex Detection (MCODE) [35] was used for analyzing modules.

## Results

### TWAS of JIA

**Fig. 2:**
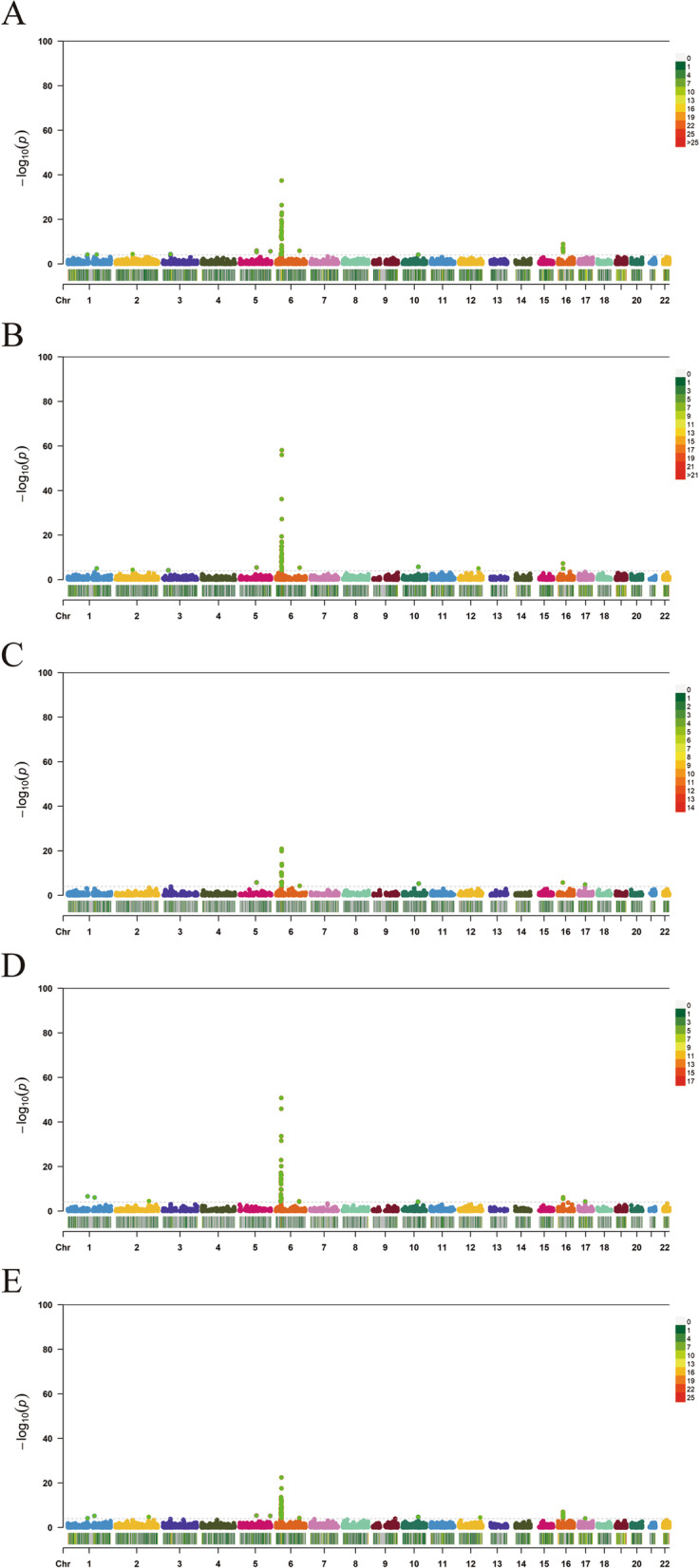
Manhattan plot of JIA-associated genes identified by TWAS (colored dots). Each dot represents a gene, the *x*-axis is the physical location (chromosomal localization), and the *y*-axis is the -log10 (*p*-value) of the gene’s association with RA. Significant genes in different tissues/cells are highlighted in different colors. **A** MS. **B** YBL. **C** NBL. **D** EL. **E** TF

**Fig. 3:**
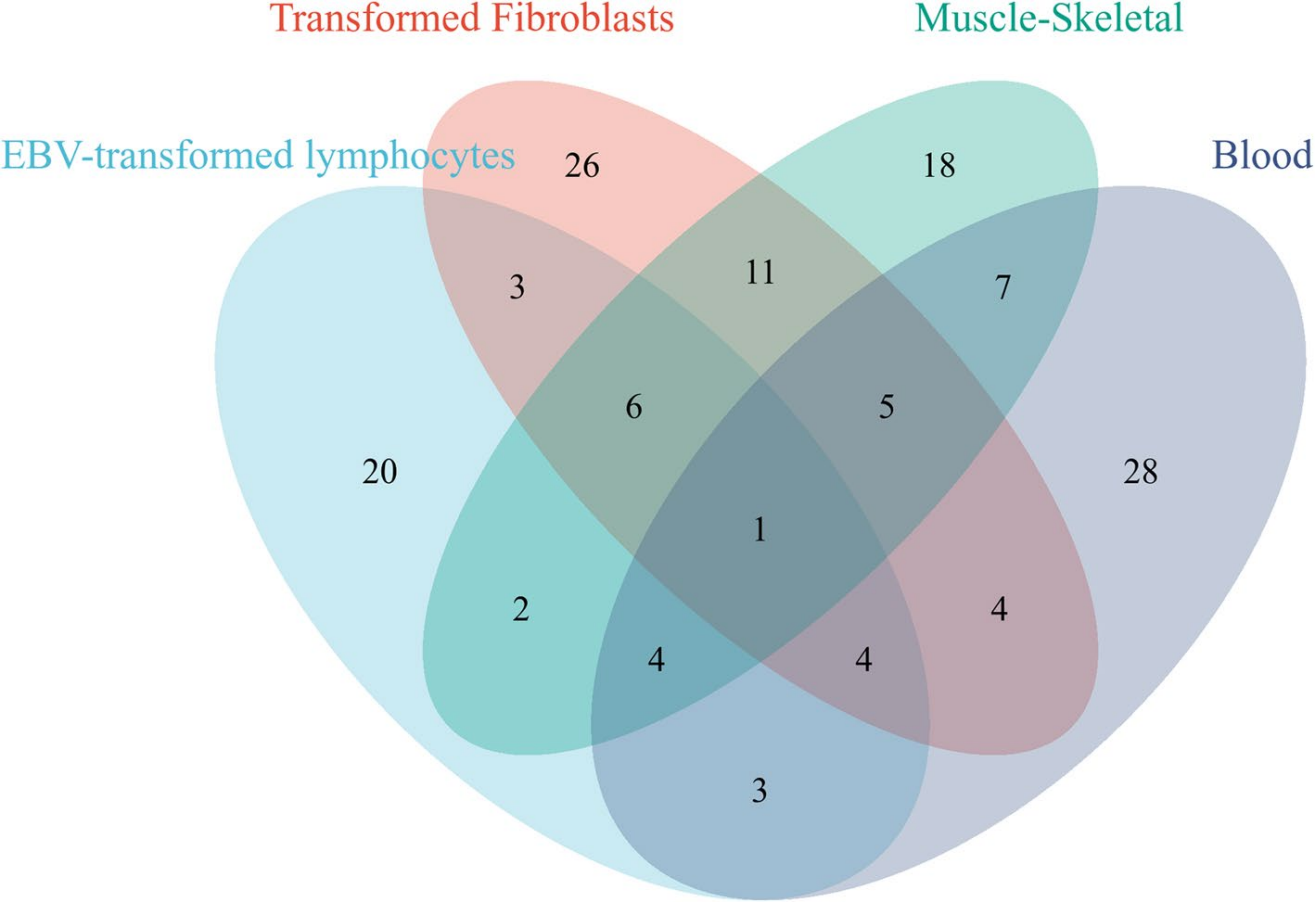
Venn diagram of genes obtained from TWAS identification in four tissues/cells. Purple, blood; blue, EL; pink, TF; green, MS

We identified 1481 genes associated with JIA by TWAS, and there were 225 genes that satisfied FDR.P < 0.05 and MODELCV.R2 ≥ 0.01, including 54, 43, 60, 24, and 44 genes expressed in muscle-skeletal (MS), EBV-transformed lymphocytes (EL), transformed fibroblasts (TF), peripheral blood (NBL), and whole blood (YBL) tissues, respectively. The genes identified by TWAS are shown in a Manhattan plot in Fig. 2. To evaluate tissue specificity as well as co-expressed genes, we performed an overlap analysis of the genes identified by TWAS in different tissues and cells, as summarized in a Venn diagram (Fig. 3). For example, 225 genes identified by TWAS were associated with JIA in TF; three genes were commonly expressed in EL and TF; four significant genes were commonly expressed in EL, TF, and blood (NTR and YFS), and the expression of one gene was common among the four joint categories. The JIA susceptibility gene jointly identified in the four tissues/cells was *HLA-DRB1*.

### Common genes identified by TWAS and mRNA expression profiling

**Fig. 4:**
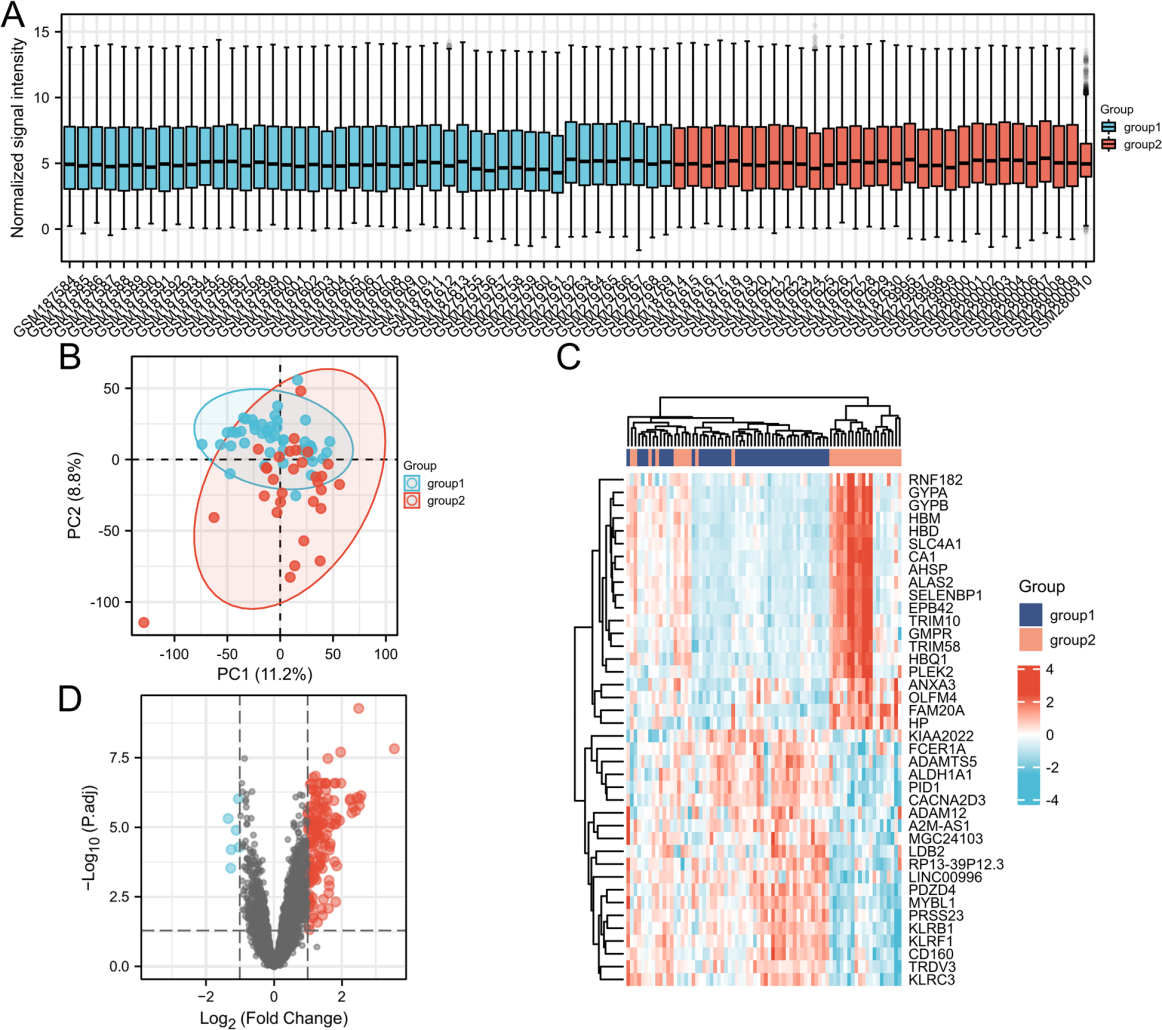
**A** The expression signal intensity of each sample detection after inter-batch difference correction, it indicates a good degree of normalization between samples. **B** The PCA plot after batch difference correction, the difference between the groups is obvious, and the subsequent analysis of variance will have more meaningful results. **C** A total of 154 differential genes were screened. **D** The top 20 gene expression of highly expressed genes versus lowly expressed genes in the results

Using |log2FC| > 1 and adjusted *p*-value < 0.05 as criteria for screening, we obtained 154 DEGs in JIA. The top 20 upregulated and downregulated genes were visualized (Fig. 4).

We compared the genes detected by TWAS and by mRNA expression profiling. The following eight common genes were identified by both analyses: *ANXA3*, *GPR146*, *KCNJ15*, *ANKRD9*, and *TMEM158*. These 8 common genes are described in Table 1.

**Table 1:** Common genes identified by TWAS in conjunction with mRNA expression profiles

Gene	Chr	BEST.GWAS.ID	TWAS.Z	TWAS.P	adj.P	logFC
ANXA3	4	rs1154761	2.08	3.78 × 10^−2^	1.00 × 10^−5^	1.94
GPR146	7	rs10246200	2.00	4.59 × 10^−2^	1.00 × 10^−5^	1.36
ANKRD9	14	rs2298877	− 2.02	4.32 × 10^−2^	2.00 × 10^−5^	1.26
TMEM158	3	rs7622843	2.22	2.66 × 10^−2^	< 1.00 × 10^−5^	1.24

*CHR* chromosome, *BEST.GWAS.ID* rsID of the most significant GWAS SNP in locus, *TWAS.Z* TWAS *Z*-score, *TWAS.P* TWAS *P* value, *adj.P* mRNA *P* value after FDR adjust

### CGSEA

We conducted a CGSEA of environmental factors and found that 287 chemical substances were significantly associated with JIA. These significant chemicals included drugs (e.g., levofloxacin), pesticides (e.g., florfenicol), herbal medicines (e.g., difenesin), phenols (e.g., nonylphenol), phthalates (e.g., dicyclohexyl phthalate), heavy metals (e.g., manganese), and air pollutants (1-nitropyrene). The top 50 compounds are listed in Table 2.

**Table 2:** The top 50 of the identified compounds

Chemical ID	Chemical name	NES	*P*
C006551	2-Amino-2-methyl-1-propanol	339.36	1.00 × 10^−4^
D002118	Calcium	15.10	1.00 × 10^−4^
C483909	Torcetrapib	12.84	1.00 × 10^−4^
D001104	Arbutin	12.17	1.00 × 10^−4^
D048628	Ketolides	7.475	1.00 × 10^−4^
C511621	2-Methyl-2H-pyrazole-3-carboxylic acid (2-methyl-4-o-tolylazophenyl)amide	6.64	1.00 × 10^−4^
D019324	Beta-naphthoflavone	6.48	1.00 × 10^−4^
D006830	Hydralazine	5.51	1.00 × 10^−4^
C051246	1-Methylanthracene	6.39	2.00 × 10^−4^
C036042	Dicyclohexyl phthalate	6.21	2.00 × 10^−4^
D007649	Ketamine	5.86	3.00 × 10^−4^
C029892	Cupric chloride	5.77	3.00 × 10^−4^
D002995	Clofibric acid	5.70	3.00 × 10^−4^
C052901	Lemongrass oil	9.05	4.00 × 10^−4^
D008713	Methimazole	7.64	5.00 × 10^−4^
D014303	Trinitrotoluene	5.61	5.00 × 10^−4^
D011345	Fenofibrate	5.30	5.00 × 10^−4^
D002065	Buspirone	4.59	5.00 × 10^−4^
D009853	Omeprazole	6.08	6.00 × 10^−4^
D008628	Mercury	5.19	6.00 × 10^−4^
D003999	Dichloroacetic acid	4.98	6.00 × 10^−4^
C029424	Hydrazine	2.72	6.00 × 10^−4^
C038753	Leptomycin B	19.31	8.00 × 10^−4^
D010081	Oxazolone	6.98	1.10 × 10^−3^
C013698	Tallow	6.52	1.10 × 10^−3^
D001507	Beclomethasone	6.28	1.10 × 10^−3^
D006160	Guanosine triphosphate	0.53	1.10 × 10^−3^
D000244	Adenosine diphosphate	5.81	1.20 × 10^−3^
C548651	2-(1′H-indole-3′-carbonyl)thiazole-4-carboxylic acid methyl ester	4.48	1.20 × 10^−3^
C001277	Geldanamycin	5.66	1.30 × 10^−3^
D002330	Carmustine	4.96	1.30 × 10^−3^
C018637	Phorone	8.24	1.50 × 10^−3^
D017239	Paclitaxel	4.03	1.50 × 10^−3^
C494474	Perchlorate	8.51	1.60 × 10^−3^
C502851	Quinocetone	6.07	1.70 × 10^−3^
C005219	Methyl cellosolve	4.69	1.70 × 10^−3^
C025256	Nonylphenol	4.35	1.80 × 10^−3^
C013418	Bromfenacoum	4.01	1.80 × 10^−3^
D000109	Acetylcholine	2.30	1.80 × 10^−3^
C032668	1-Nitropyrene	6.28	1.80 × 10^−3^
D008274	Magnesium	3.51	2.00 × 10^−3^
D010672	Phenytoin	4.87	2.10 × 10^−3^
D020001	1-Butanol	3.92	2.10 × 10^−3^
C019499	2-Nitrofluorene	6.20	2.20 × 10^−3^
C002741	N-nitrosomorpholine	4.57	2.20 × 10^−3^
C016151	Tinuvin	4.40	2.20 × 10^−3^
D008383	Margarine	1.49	2.20 × 10^−3^
C576882	1-(2-Trifluoromethoxyphenyl)-2-nitroethanone	5.70	2.30 × 10^−3^
D020245	p-chloromercuribenzoic acid	4.12	2.30 × 10^−3^

### Functional exploration of the TWAS-identified genes associated with JIA

**Fig. 5:**
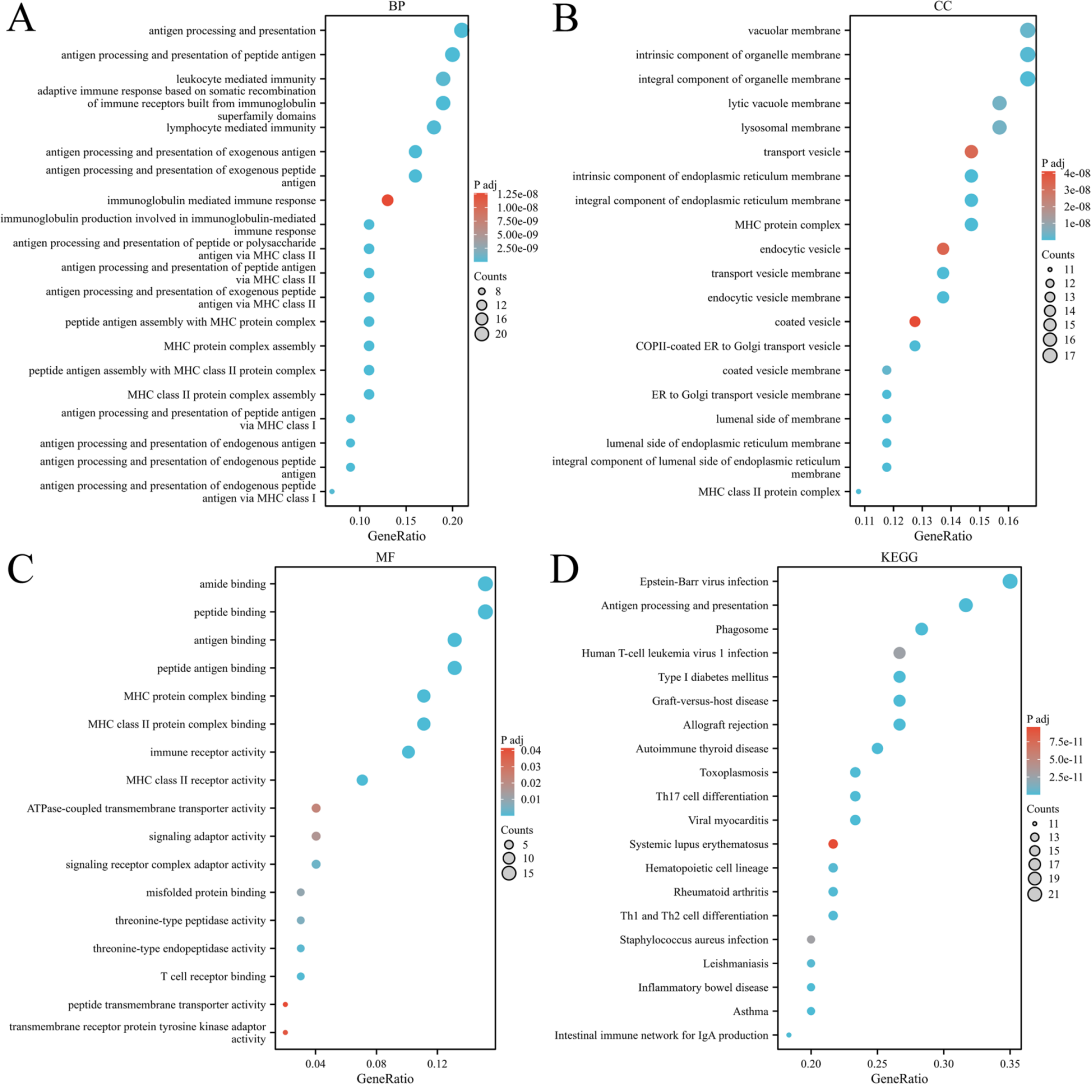
Network diagram of GO term analysis for TWAS-identified genes, where each circular point in the network represents a term whose size is proportional to the number of input genes for that term. **A** BP. **B** CC. **C** MF. **D** KEGG

**Fig. 6:**
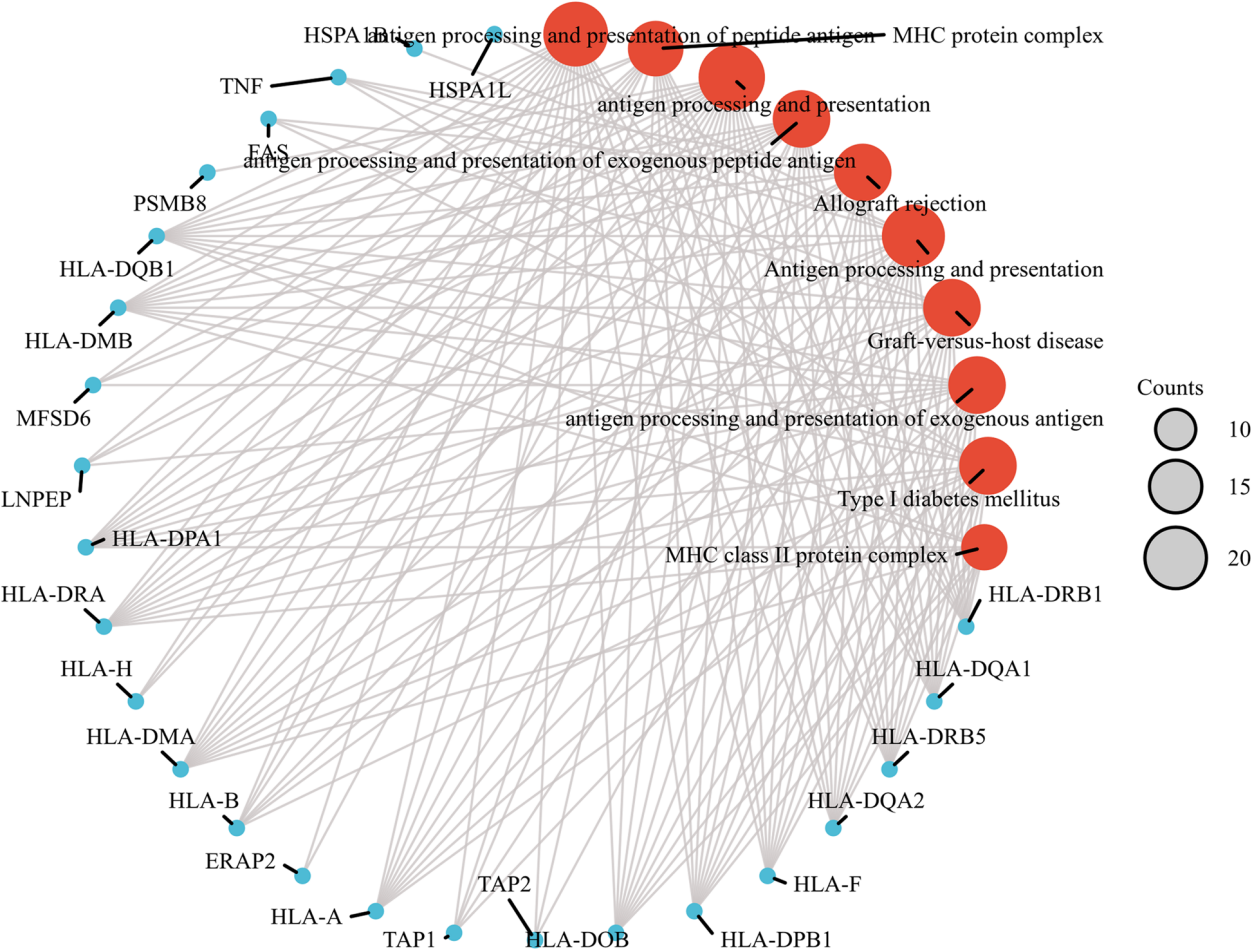
Network diagram of the top 10 pathways with the smallest p.adjust

We performed GO and KEGG pathway enrichment analyses of 225 genes identified by TWAS and detected 267 GO terms and 37 KEGG terms. Next, 179 GO terms and 36 KEGG terms were screened with p.adjust < 0.05, such as antigen processing and presentation of peptide antigen via MHC class I, T-cell–mediated cytotoxicity, rheumatoid arthritis, and human T-cell leukemia virus 1 infection. The results are shown in Fig. 5. The top 10 pathways with the lowest p.adjust are summarized in Fig. 6, such as the T-cell receptor signaling pathway and MHC class II protein complex.

### Protein–protein interaction network analysis

**Fig. 7:**
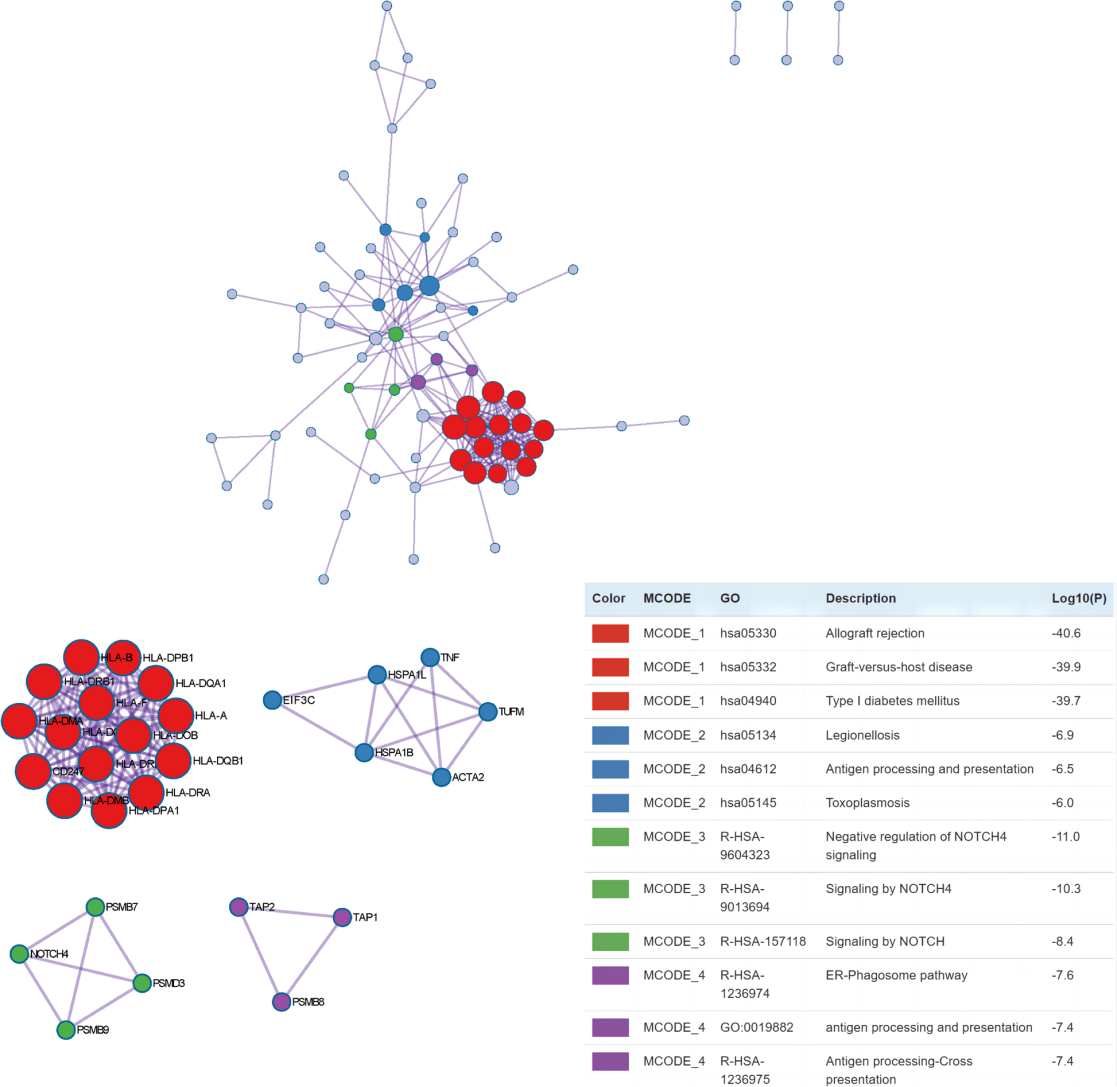
The PPI of AS-associated proteins

To identify densely connected regions in the PPI network, we formed eight MCODE clusters with PPI network genes (Fig. 7). The hub genes identified using the MCODE plugins were further evaluated by functional analyses. For example, MCODE1 was associated with autoimmune diseases, MCODE2 was associated with legionellosis and antigen processing presentation, and MCODE3 was associated with negative regulation of NOTCH4 signaling.

## Discussion

GWAS is a common method for the screening and identification of candidate genes involved in complex diseases. However, most loci identified by this approach are located in the non-coding regions, making it difficult to explain the relative risk [36]. Therefore, to understand JIA pathogenesis, we performed TWAS using large-scale GWAS data. The main symptoms of JIA are inflammation of the joints as well as extra-articular manifestations, including fever, enlarged lymph nodes, rash, and plasmacytosis [37]; various immune events occur not only in the joints but also on extra-articular mucosal surfaces and primary lymphoid tissues, especially the synovium. Thus, several types of tissues and cells are affected, including the synovial membrane, cartilage, bone, fibroblasts, adipocytes, macrophages, and immune cells [38]. Based on a previous TWAS of RA by Wu et al., we selected MS, EL, TF, NBL, and YBL tissues as gene expression references [38]. We combined the TWAS results with mRNA expression data for JIA to identify candidate genes and performed a pathway enrichment analysis for these genes. Finally, we performed a CGSEA using the pooled TWAS data to identify the environmental factors and chemicals that may be associated with the pathogenesis of JIA.

Integrating TWAS and mRNA expression profiling data revealed several candidate genes associated with JIA, and compared to previous studies, in the present study, we identified some novel genes that may play a potential role in the pathogenesis of JIA, such as *ANXA3*, *GPR146*, *ANKRD9*, and *TMEM158*. Reportedly, *ANXA3* contributes to cancer development via the NF-κB pathway [39]. NF-κB and RANK ligand receptor activator expression in the joints of children with JIA may facilitate the survival of inflammatory cells in the joints [40]. Some studies have shown that *GPR146* deficiency reduces lipids and prevents atherosclerosis [41], and the study by Clarke et al. found that genetic susceptibility to juvenile idiopathic arthritis is associated with multiple cardiovascular risk factors [42], supporting the hypothesis of increased cardiovascular risk in juvenile idiopathic arthritis, suggesting that *GPR146* may be associated with the development of JIA. *TMEM158* was initially reported as a Ras-induced gene during aging and classified as an oncogenic or tumor suppressor depending on the tumor type [43]. The potential mechanisms involving STAT3 activation mediating TMEM158-driven glioma progression have also been identified, and the inhibitory effect of *TMEM158* downregulation on glioma growth has been confirmed [44]. While autoimmune diseases have many similarities with cancer, there are many links and similarities in the pathogenesis of both, so we speculate that *TMEM158* may be closely related to the development of JIA.

GO analyses revealed enrichment for several terms, such as T-cell–mediated immune regulation, interferon-gamma–mediated signaling pathway, neutrophil activation, processing and expression of endogenous peptide antigens via MHC class I, processing and expression of antigens via MHC class II, autoimmune thyroid diseases, and rheumatoid arthritis. JIA is characterized by a loss of immune tolerance, and it is believed that the balance between the activity of effector T and regulatory T-cells in the joints is disturbed, leading to the chronic inflammation of the joints and JIA [45]. Our results demonstrate the importance of T-cell–mediated immunity in the pathogenesis of JIA. A life-threatening complication of systemic JIA (SJIA), macrophage activation syndrome (SJIA-MAS), is characterized by a cytokine storm and dysregulated T-lymphocyte proliferation [46]. Our results also confirm that interferons may be involved in JIA development. Several studies have linked antigen processing and expression via MHC class I and MHC class II to the development of JIA, and a recent large-scale study identified the MHC locus as the strongest genetic risk region for JIA [47].

We used an extended classical GSEA with a large-scale GWAS aggregate dataset to detect the associations between environmental chemical substances and JIA and identified 281 chemical substances, including drugs, organic compounds, inorganic compounds, plants extracts, nutrients, phenols, air pollutants, and heavy metals. As a common component used in many consumer products, 2-amino-2-methyl-1-propanol is a promising amine for use in industrial-scale post-combustion CO, as well as being an atmospheric pollutant [48]. Yavorskyy et al. detected high levels of 2-amino-2-methyl-1-propanol in the synovial fluid of osteoarthritic knees [49]; thus, it may be related to the onset of joint inflammation to some extent, corroborating our findings. Arbutin is a plant extract often present in skin care products owing to its whitening effect; there is evidence for a combined effect of arbutin and indomethacin on inducing inflammation [50]. As a widespread environmental contaminant with many toxic effects, including roles in endocrine disruption, reproductive dysfunction, immunotoxicity, liver damage, and cancer, 2-methyl-2*H*-pyrazole-3-carboxylic acid amide may contribute to the development of JIA to some extent [51]. β-Naphthoflavone is present in cigarette smoke condensate, and Adachi et al. found that cigarette smoke condensate can lead to AhR-dependent NF-κB activation and activate related pathways, thereby inducing the production of the pro-inflammatory factor IL-1β in synoviocytes of patients with rheumatoid arthritis [52]. This is consistent with our findings indicating that beta-naphthoflavone is associated with JIA onset.

In this study, we combined TWAS and mRNA expression profiling to identify the candidate genes and biological pathways related to JIA. The combination of these two methods can accurately identify candidate genes. We performed functional enrichment and PPI network analyses of the identified genes and determined biological processes associated with JIA pathogenesis. Finally, we identified several environmental chemicals that may be associated with JIA. Our results provide new insights into the pathogenesis of JIA and its risk factors.

## Limitations of the study

The limitations of this study include the following: First, the pooled GWAS data were obtained from the UK Biobank, and the study subjects were mostly from European populations. Thus, the results of this study may not be generalizable to other populations. Second, some candidate genes for JIA obtained have not been validated by molecular biology experiments; these genes should be evaluated by functional assays in future research.

## Conclusion

In summary, we integrated the GWAS dataset of JIA from the UK Biobank to complete TWAS. Then, we further compared the genes identified by TWAS with those identified by mRNA expression profiling and performed GO and KEGG analyses and PPI network construction to identify genes and biological pathways associated with the pathogenesis of JIA. Finally, we performed CGSEA analysis to obtain chemical substances and environmental factors associated with the pathogenesis of JIA. Our results provide a new direction for the study of the mechanisms of JIA at the genetic and molecular levels and new ideas for the chemical environmental factors associated with JIA.

## Supplementary Information

**Additional file 1:.** TWAS results.

**Additional file 2:.** mRNA results.

**Additional file 3:.** CGSEA results.

## Data Availability

The datasets analyzed during the current study are available from the Gene Expression Omnibus database (https://www.ncbi.nlm.nih.gov/gds) accession number: GSE7753 and GSE11083, and the UK biobank (http://geneatlas.roslin.ed.ac.uk/) fields: 20002.

## Abbreviations

JIA: Juvenile idiopathic arthritis
RA: Rheumatoid arthritis
EL: EBV-transformed lymphocytes
TF: Transformed fibroblasts
NBL: Peripheral blood
YBL: Whole blood
GWAS: Genome-wide association study
TWAS: Transcriptome-wide association studies
eQTL: Expression quantitative trait loci
DEGs: Differentially expressed genes
GTEx: Genotype-Tissue Expression
PPI: Protein–protein interaction
GO: Gene Ontology
KEGG: Kyoto Encyclopedia of Genes and Genomes
